# Dealing with the Sac in the Radical Cure of Inguinal and Femoral Hernia

**Published:** 1912-01

**Authors:** 


### Dealing With the Sac in the Radical Cure of Inguinal and Femoral Hernia.

There is reason to believe, says H. McClure Young of St. Louis, that the ordinary indirect inguinal hernia is always to that extent congenital that a sac lined with peritoneum persists after the descent of the testicle is accomplished, and that this sac awaits only some extraordinary exertion or some relaxed condition of the parts to receive a loop of bowel from above. In dealing with hernia, therefore, the obliteration of this sac must always be insisted upon as the one all-important step in the operation. From this standpoint Young discusses the various operations for inguinal hernia, but says that none so logically answers the necessities of the condition as the one devised by Lexer, which he describes as follows: The skin and aponeurosis of the external oblique are divided in the usual way and the sac freed as far up as the internal ring, where it is ligated securely as high up as possible, but not yet removed. A pair of slightly curved forceps is now passed under the free margin of the conjoined tendon, insinuating them gently upward between the muscle and peritoneum for a distance of about two inches. Here the point of the forceps is pushed forward through the muscle. Into the jaws of this forceps is now introduced the jaws of a second pair of similar forceps, locking them securely and withdrawing the first pair, thus conducting the second pair along the route of the first down toward the internal ring. The loose end of the sac is now clasped in the jaws of the forceps which have been thus placed, and the forceps withdrawn. This brings the sac out through the muscular tissue at a point about two inches above the internal ring. It is pulled upon until the neck of the sac or point of original ligature comes to lie firmly against the posterior surface of the muscle at this point, a thing which requires no great amount of force. Two or three sutures now anchor the sac to the muscle and the redundant portion of the sac is cut away. The Bassini operation may now be performed or any other procedure resorted to which the requirements of the case may seem to indicate. Should the surgeon wish to avoid drawing the sac through the muscular tissue, he may proceed as follows: Having ligated the sac, he leaves the end of his ligatures long and threads each upon a needle. He then removes the sac, and passes an additional suture through the neck of the sac and again threads each end upon a needle. He now inserts a finger under the free margin of the conjoined tendon and dissects it bluntly from the peritoneum for a distance of about two inches, at which point he passes his needles through the muscular tissue from within outward in such manner that his knots when tied shall lie in a direction parallel with the muscular fibers and about a centimetre and a half apart. The tying of these knots now draws the neck of the sac firmly up against the posterior surface of the muscle. The author says that when surgeons in general understand more perfectly the object aimed at and always to be kept in mind in such operations, the old practice of leaving the neck of the sac at the mouth of the hernial opening to invite recurrence will become obsolete.—*Interstate Medical Journal*, October, 1911.

